# Gas6 protein: its role in cardiovascular calcification

**DOI:** 10.1186/s12882-016-0265-z

**Published:** 2016-05-26

**Authors:** Nadine Kaesler, Svenja Immendorf, Chun Ouyang, Marjolein Herfs, Nadja Drummen, Peter Carmeliet, Cees Vermeer, Jürgen Floege, Thilo Krüger, Georg Schlieper

**Affiliations:** Uniklinik RWTH Aachen, Nephrology, Aachen, Germany; University of Maastricht, R&D Group, Maastricht, Netherlands; University of Leuven, Vesalius Research Center, VIB, Leuven, Belgium

## Abstract

**Background:**

Cardiovascular calcifications can be prevented by vitamin K and are accelerated by vitamin K antagonists. These effects are believed to be mainly mediated by the vitamin K-dependent matrix Gla protein. Another vitamin K-dependent protein, Gas6, is also expressed in vascular smooth muscle cells (VSMC). In vitro Gas6 expression was shown to be regulated in VSMC calcification and apoptotic processes.

**Methods:**

We investigated the role of Gas6 in vitro using VSMC cultures and in vivo in young and old Gas6-deficient (Gas6^-/-^) and wildtype (WT) mice. In addition, Gas6^-/-^ and WT mice were challenged by (a) warfarin administration, (b) uninephrectomy (UniNX) plus high phosphate diet, or (c) UniNX plus high phosphate plus electrocautery of the residual kidney.

**Results:**

In vitro VSMC from WT and Gas6^-/-^ mice exposed to warfarin showed increased apoptosis and calcified similarly. In vivo, aortic, cardiac and renal calcium content in all groups was similar, except for a lower cardiac calcium content in Gas6^-/-^ mice (group a). Von Kossa staining revealed small vascular calcifications in both WT and Gas6^-/-^ mice (groups a-c). In aging, non-manipulated mice, no significant differences in vascular calcification were identified between Gas6^-/-^ and WT mice. Gas6^-/-^ mice exhibited no upregulation of matrix Gla protein in any group. Cardiac output was similar in all treatment groups.

**Conclusions:**

Taken together, in our study Gas6 fails to aggravate calcification against the previous assumption.

## Background

Cardiovascular calcifications are highly prevalent in chronic kidney disease and are associated with an increased morbidity and mortality [1]. They can be accelerated by warfarin, a direct inhibitor of the vitamin K regenerating cycle [2]. Vascular calcifications occur in the arterial media and intima [3]. Vascular smooth muscle cells (VSMC) of the arterial media express two vitamin K-dependent proteins, gla rich protein [4], matrix Gla protein (MGP) [5] and Gas6 [6]. Both require reduced vitamin K (KH_2_) as a cofactor for posttranslational γ-carboxylation and activation. MGP potently inhibits vascular calcification via interference with hydroxyapatite crystal formation [7].

In contrast to MGP, the role of Gas6 in vascular calcification has repeatedly been suggested [8, 9] but so far has remained largely speculative. Gas6 shows 40 % homology to protein S; both are members of the vitamin K family. Protein S is predominantly expressed in the liver [10] whereas Gas6 is highly expressed in the kidney, heart and lungs [11]. Both proteins are ligands for the Axl tyrosine kinase receptors. These receptors regulate cell survival and apoptosis [12]. Vitamin-K-dependent carboxylation of Gas6 is essential for its binding to the Axl receptor [13]. Tyrosine phosphorylation of Axl induces cell proliferation [14]. Gas6 is known to protect endothelial cells and VSMC against apoptosis [15, 16], and apoptotic bodies are known to be associated with vascular calcifications. Another potential and more coherent link between Gas6 and vascular calcifications are in vitro data showing that phosphate-induced calcification of VSMC is associated with a downregulation of Gas6 expression [16]. In addition, antiapoptotic effects and protection of calcification of VSMC by statins were apparently mediated through Gas6 mRNA stabilization [16]. Also effects of testosterone [9], taurine [17] and adiponectin [18] in cells were linked to alterations in Gas6 expression. Binding of Gas6 by alpha lipoic acid resulted in decreased apoptosis and calcification in VSMC and mice [8]. So far no in vivo data are available on the role of Gas6 itself in cardiovascular calcification. To clarify this, we assessed Gas6 knockout (Gas6^-/-^) mice and Gas6^-/-^ derived VSMC in in vitro and in vivo in cardiovascular calcification models.

## Methods

### Animals & diets

**Fig. 1:**
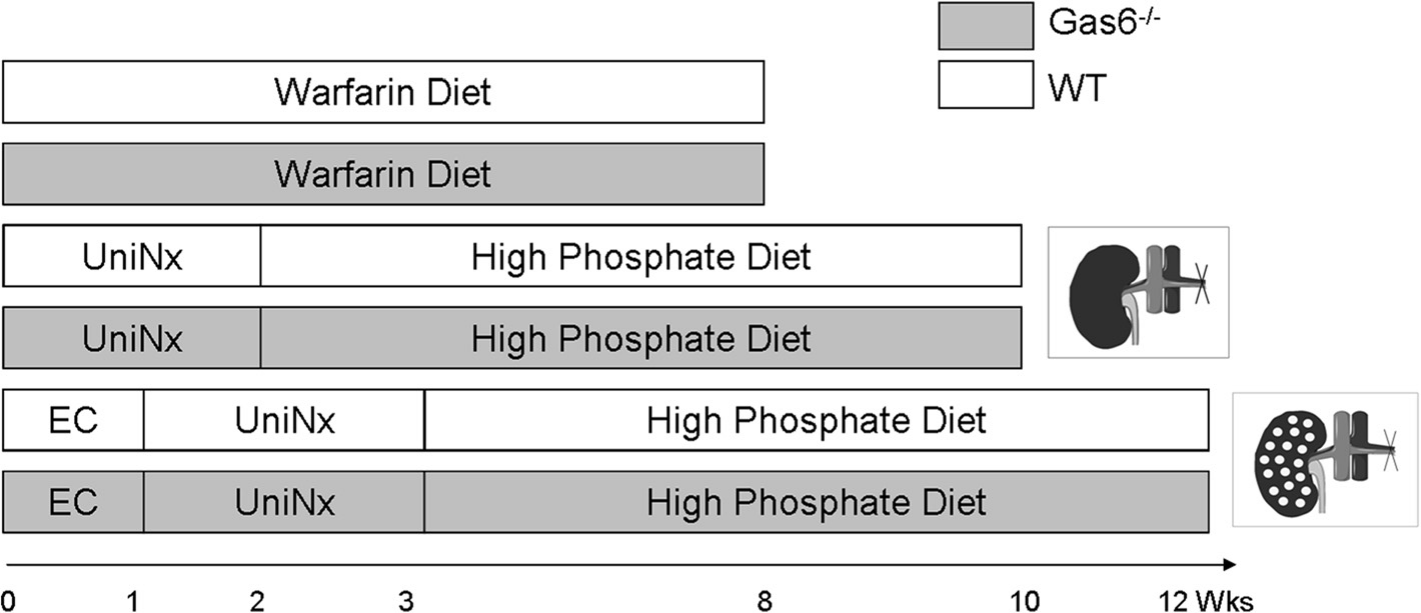
Experimental design of the in vivo experiments. Ten WT or Gas6^-/-^ mice received warfarin diet for 8 weeks or a uninephrectomy combined with a high phosphate diet or uninephrectomy in combination with electrocauterization of the contralateral kidney together with high phosphate diet

Gas6^-/-^ mice, as described previously [19], were backcrossed for more than 10 generations onto a C57BL/6 background. They received feed and water *ad libitum*. Healthy, aging mice were sacrificed at the age of 34–36 weeks. For the surgical groups (uninephrectomy, UniNx or electrocautery) the rodent chow (AB diets, Woerden, the Netherlands) was supplemented to 0.95 % calcium and 1.65 % phosphate. The warfarin chow contained 3 g/kg. The warfarin diet or surgery was started at the age of 8–10 weeks and the diets were continued for 8 weeks. UniNx was performed at the age of 8 weeks. Therefore, the left kidney was eventrated and -after ligation of the ureter with silk - fully removed. After one week of recovery, the high phosphate diet was initiated. For more severe kidney damage, electrocautery of the right kidney at the age of 8 weeks plus UniNx of the left kidney 2 weeks later were performed. Punctual lesions were set on the renal cortex with a 2-mm diameter electrocoagulation ball tip over all areas of the kidney (Erbe, Erbotom ACC 450, Tübingen, Germany). The 1-mm deep punctuate lesions were spaced 2 mm apart [20]. After 2 weeks, the contralateral kidney was removed and after one additional week the high phosphate diet was started (Fig. 1). In all experiments, only female animals were used, except in aging mice where both genders were analyzed (age matched). In our in vivo models 10 animals per group were used at least, except in the electrocautery group where only 4 animals could be analysed because of high mortality and 6 animals for the initial, measurements in healthy mice. All animals were treated in accordance with the requirements of the Federation of the European Laboratory Animals Science Associations. The protocol was approved by the Landesamt für Natur, Umwelt und Verbraucherschutz NRW, Germany (Permit Number 84-02.04.2011.A144). All surgery was performed under ketamine/rompun anaesthesia and all efforts were made to minimize suffering.

### VSMC cell culture

VSMC were isolated from the thoracic aorta of C57BL/6 and Gas6^-/-^ mice. After harvesting, aortas were incubated with 2 g/L collagenase, 1 % elastase for 1 h at 37 °C. The cell culture medium (PromoCell, Heidelberg, Germany) was supplemented with 1 % penicillin streptomycin and 0.1 % gentamycin. Passages from 3–5 were used for calcification experiments. Calcification medium contained 3-mM calcium phosphate or 10 nM, 3 m M calcium phosphate and 10 μM warfarin or 3 and 8 mM beta glycerolphosphate (BGP) plus 3 mM calcium (all chemicals from Sigma Aldrich, Munich, Germany). To induce calcification, cells were cultured for 5 to 7 days with calcification medium. A negative control (d_0_) was included in each setup to normalize the obtained values and calcium deposits were depicted as mg/g total protein.

### Cardiovascular parameters

Transthoracic echocardiography was performed on the Vevo 770 (Visualsonics, Ontario, Canada). Therefore, mice were anesthetised with isoflurane (Abbott, IL, USA) and placed on a warming plate at 37 °C. Breathing and heart frequency were monitored continuously. Left ventricular (LV) mass was calculated in the M-mode in the long axis view from the Devereux formula. Ejection fraction (EF) as an index for global left ventricular systolic function were measured by the Simpson's method. Stroke volume was estimated as the difference between the LV end-systolic and end-diastolic volumes. Pulse-wave velocity in the right common carotid artery and the abdominal aorta were measured using the transit-time method in a two-dimensional mode. In the carotid artery, the proximal pulse-wave signal was obtained 1 mm behind the origin of the subclavian artery, the distal signal 1 mm before the carotid bifurcation. The transit time was found by subtracting the distal arrival time between the ECG R-wave peak and the foot of velocity upstroke from the similarly determined proximal arrival time [PWV = Δd / (Pt_dist_ – Pt_prox_)], where Pt is the time point of the proximal or distal pulse- wave signal and Δd is the distance between the two measurements.

### Biochemistry

Blood was collected by puncture of the left ventricle, terminally. After sedimentation, serum was obtained by centrifugation at 2000 × g for 10 min. Urine was collected for 24 h before sacrifice. Serum and urine parameters were measured by clinical routine laboratory diagnostics (Vitros 250, Ortho Clinical Diagnostics, NY, USA).

Protein content was measured by the Pierce Bicinchoninic Acid method, as described by the manufacturer (Thermo Fisher Scientific, IL, USA) [21].

Additionally, the murine serum was tested by total ucMGP ELISA (VitaK BV, Maastricht, the Netherlands) [22] and protein S ELISA Kit (antibodies online, ABIN628120, Aachen, Germany).

### RT-PCR

Genotyping of knockout animals and VSMCs was performed as described previously [23].

RNA was isolated from RNAlater stabilized aortic tissue (liver, abdominal aorta) (QIAGEN Rneasy, Hilden, Germany). Purity and RNA concentration were analyzed with the Agilent RNA 6000 Nano Kit (Agilent, Böblingen, Germany). The reverse transcriptase was performed by the Reverse Transcriptase Core Kit (RT-RTCK-05, Eurogentec, Cologne, Germany). Quantification of MGP gene expression was performed on an Applied Biosystems 7500 Real-Time PCR TagMan® system with external standards for MGP (8.8 * 10^6^ – 8.8 * 10) and GAPDH (6.4 * 10^6^ – 6.4 * 10). MGP probe was 5’AGAGTCCAGGAACGCAACAAGCCTGC 3’, sense primer 5’ GCAGAGGTGGCGAGCTAAAG 3’ and antisense primer 5’ AGCGCTCACACAGCTTGTAGTC 3’. Cbfa1 sense primer was 5’ CAAGTAGCCAGGTTCAACGATCT 3’, cbfa1 antisense 5’ GACTTGTATGGTCAAGGTGAAACTCTT 3’; OPN sense 5’ GACCATGAGATTGGCAGTGATTT 3’, OPN antisense 5’ GATCTGGGTGCAGGCTGTAAAG, Axl sense 5’ TCTGGCTGGGAAAGTCAGAT 3’, Axl antisense 5’ CAGCCGAGGTATAGGCTGTC 3’ [24]. GAPDH probe was 5’ AAGGCCGAGAATGGGAAGCTTGTCATC 3’, sense primer 5’ AAGTGGTGATGGGCTTCCC 3’ and antisense primer 5’ GGCAAATTCAACGGCACAGT 3’.

### Calcium measurement

Tissue calcium content was measured with the cresolphthalein assay. Tissues (kidney, heart, aortic arch) were lyophilisated on a Christ Loc 1mALPHA 1-4 (Martin Christ, Osterode am Harz, Germany) coupled with a vacuum hybrid pump RL 6 (Vacuubrand, Wertheim, Germany). Calcium was mobilised in 10 % formic acid and quantified by the cresolphthalein method (Randox Laboratories, Crumlin, UK). Absorption was measured at 550 nm on a Tecan Sunrise microplate absorbance reader (Tecan, Mennedorf, Switzerland).

### Histochemistry

Localisation of phosphate crystals was analyzed by *von Kossa* staining [25, 26] (kidney, heart base, descending aorta). Sirius red staining was performed in heart tissues by 5 % (w/v) Sirius red (Sigma Aldrich, Munich, Germany) in picric acid (Sigma Aldrich, Munich, Germany) followed by washing in acidified ethanol (70 % (v/v); pH 3.5). Apoptosis measurements were performed with the in situ cell death detection kit (Roche, Ref. 11684817910, Basel, Switzerland) according to the manufacturer’s protocol. Cells were counterstained with DAPI (Vectashield, Vector Laboratories, CA, USA). Apoptosis was quantified by counting TUNEL positive VSMCs and by planimetric analysis in aortic sections (Keyence BZ-II Analyzer, Neu-Isenburg, Germany).

### Statistics

Differences between treatment groups were assessed by one-way ANOVA followed by Tukey’s multiple comparison test. Equal variances were tested with Bartlett’s method. D’Agostino and Pearson normality test was performed to check for Gaussian distribution. Statistical significance was defined as *p* < 0.05, highly significant *p* < 0.001. Comparison between only two groups was performed by Students *t*-test in WT and knockout animals after different treatment periods.

## Results

### In vitro VSMC calcification

**Fig. 2:**
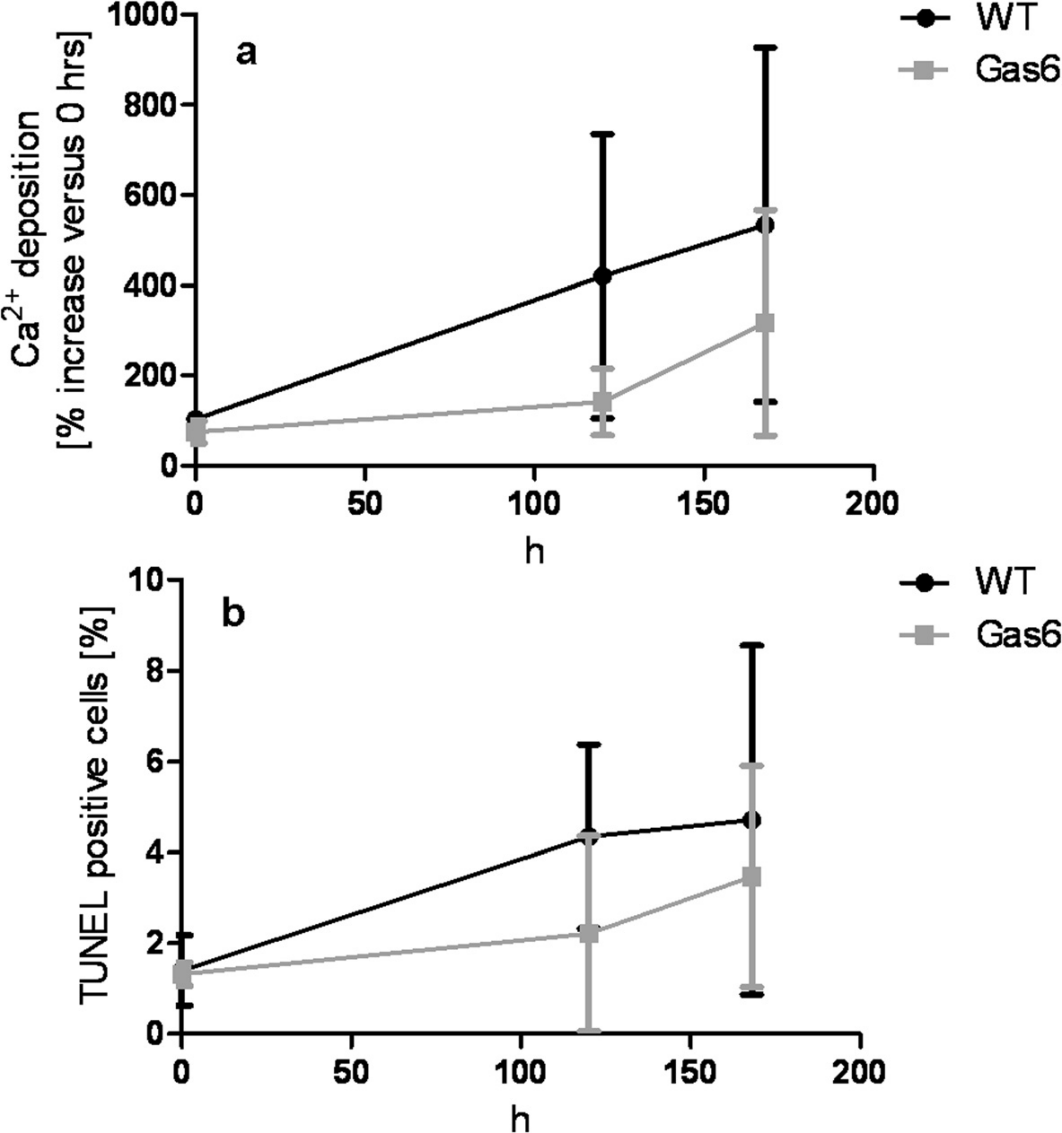
Calcium deposition **a** Ca^2+^ deposition in VSMC derived from Gas6^-/-^ and WT mice after 168 h (h) of exposure to phosphate and calcium-enriched cell culture medium. **b** TUNEL-positive VSMC of Gas6 and WT mice after exposure to phosphate and calcium-enriched cell culture medium. VSMC (Vascular Smooth Muscle Cells), WT (Wildtype); mean ± SD; *: the increase in apoptosis was significant for WT cells after 120 h and 168 h compared to 0 h (*p* < 0.05); ** the increase in calcification was highly significant for WT cells after 168 h compared to 0 h (*p* < 0.001)

In vitro, VSMC of WT and Gas6^-/-^ mice were challenged by calcification medium or by warfarin. Using calcification medium, the deposition of calcium in VSMC increased over time: though in early stages not significant, after 120 h the calcium content had increased 4-fold in WT and 1.4-fold in Gas6^-/-^ VSMC (Fig. 2a). After 168 h of incubation, the calcium load of VSMC increased further (significant for WT cells compared to 0 h). There was no significant difference between VSMC from Gas6^-/-^ and WT mice (Fig. 2).

**Fig. 3:**
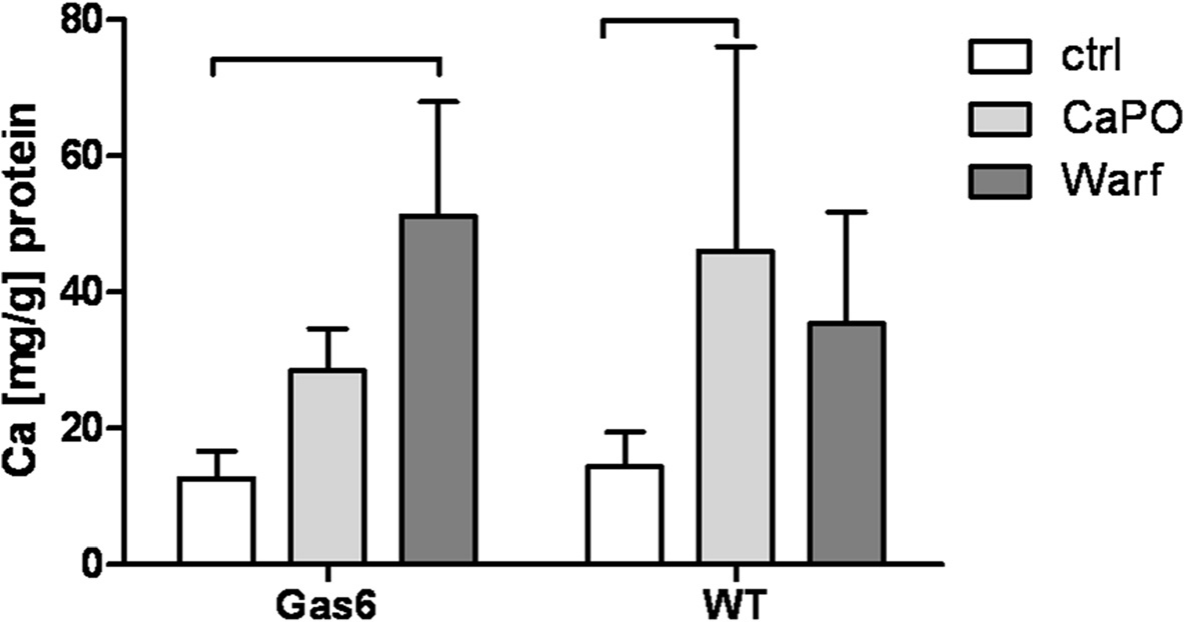
Calcium deposition a) Ca^2+^ deposition in VSMC isolated from WT and Gas6^-/-^ mice after treatment with calcium phosphate and warfarin over 5 days; mean ± SD. *: *p* < 0.05; **: *p* < 0.001

Warfarin treatment at a low dosage of 10 nM significantly increased calcium deposition in WT cells after 120 h (WT t_120_: + 648 % ± 540 % change in calcium deposition). Again, we could not observe a significant difference in calcium load between Gas6^-/-^ and WT-derived VSMC (data not shown). Calcium phosphate load led to an increased calcium deposition in WT cells and the highest dosage of 10 μM of warfarin significantly increased calcium deposition after 120 h in Gas6^-/-^ cells (Fig. 3).

Treatment with 3 mM BGP significantly increased calcium deposits only in WT-VSMC after 120 h. Ca-deposition at 8 mM BGP was similar in Gas6^-/-^ and WT VSMC (data not shown).

The rate of VSMC apoptosis in calcification medium increased significantly at 120 h and 168 h in WT cells. The rate of apoptosis was not different in Gas6^-/-^ compared to WT VSMC (Fig. 2b).

### In vivo calcification models

#### Young healthy mice

In vivo, we first compared untreated animals of both groups (Gas6^-/-^ and WT). At the age of 8 weeks, out of a number of serum parameters, only alkaline phosphatase was lower in healthy Gas6^-/-^ than in WT mice (Table 1). Echocardiography revealed a lower LV mass in healthy WT mice compared to healthy Gas6^-/-^ at the age of 8 weeks (Table 1).

**Table 1:** Basal biochemistry and echocardiography

	8–9 weeks old, healthy		34–36 weeks old, healthy	
Parameter	C57BL/6	Gas6^-/-^	C57BL/6	Gas6^-/-^
Body weight [g]	20.2 ± 0.7	18.6 ± 1.6	30.1 ± 6.4	26.4 ± 4.2
*Serum parameter*				
Urea [mmol/l]	10.8 ± 3.3	11.6 ± 1.9	6.6 ± 1	6.0 ± 0.9
Creatinine [μmol/l]	19.6 ± 2.1	24.5 ± 8.6	18.0 ± 5.9	26.2 ± 16.7
Calcium [mmol/l]	2.48 ± 0.10	2.23 ± 0.08	2.39 ± 0.13	2.39 ± 0.09
Phosphate [mmol/l]	3.11 ± 0.91	3.05 ± 0.82	2.88 ± 0.45	2.85 ± 0.34
Protein [g/dl]	4.46 ± 0.74	4.90 ± 0.15	6.00 ± 0.42	5.50 ± 0.26*
Alkaline phosphatase [U/l]	255 ± 106	256 ± 35	142 ± 54	207 ± 69
ucMGP [nM]	3158 ± 458	2987 ± 554	3523 ± 1645	3532 ± 1095
Protein S [ng/ml]	0.97 ± 0.24	0.87 ± 0.16	0.80 ± 0.14	0.72 ± 0.14
*Echocardiography*				
Left ventricular mass [mg]	50.8 ± 11.4	73.6 ± 9.8*	98.6 ± 15.2	81.8 ± 29.5
Stroke volume [μl]	23.9 ± 9.0	20.3 ± 6.3	23.6 ± 6.2	19.8 ± 4.3
Ejection Fraction [%]	54.3 ± 11	45.6 ± 7.8	40.1 ± 9.6	41.5 ± 11.0
VACC [mm/ms]	2.09 ± 0.98	2.06 ± 1.15	2.13 ± 1.52	1.72 ± 0.72

Baseline biochemical and functional characteristics of healthy wildtype and Gas6^-/-^ mice at different ages (Mean ± SD), *ucMGP* uncarboxylated matrix Gla protein, *V*_*ACC*_ pulse-wave velocity over the common carotid artery, *WT* wildtype *p < 0.05 to corresponding wildtype control

**Fig. 4:**
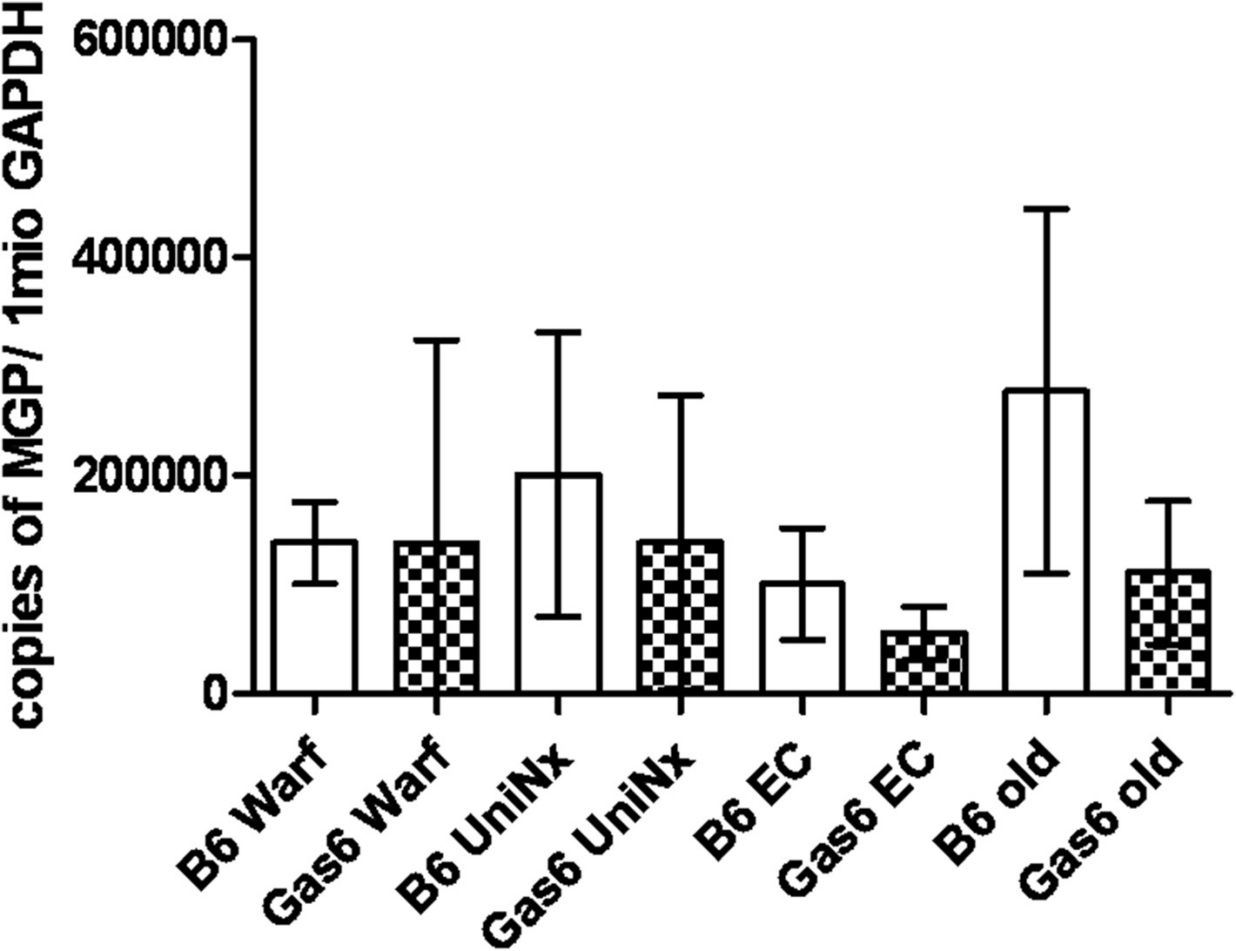
MGP expression; MGP mRNA expressed as copies of MGP per 1 million copies of GAPDH measured by RT-PCR; mean ± SD

**Fig. 5:**
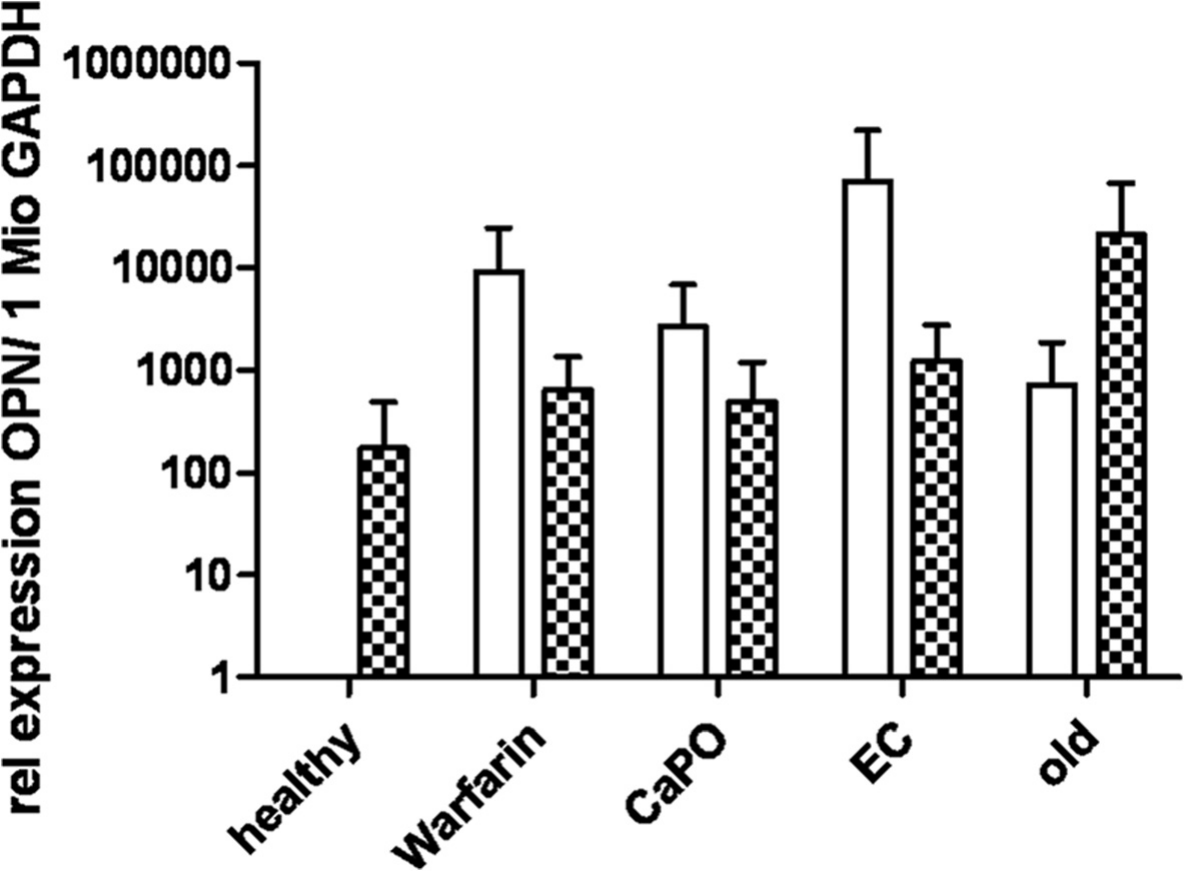
Osteopontin expression; Osteopontin mRNA expressed as relative expression per 1 million copies of GAPDH measured by RT-PCR; mean ± SD

We next tested whether in Gas6^-/-^ mice upregulation of the calcification inhibitor MGP occurred. However, in Gas6^-/-^ mice MGP gene expression and serum undercarboxylated MGP (ucMGP) levels were equal to WT (Fig. 4). Similarly, serum levels of circulating protein S, which shows a 43 % homology with Gas6 [10], did not differ between the mouse strains (Table 1). Expression of the osteoblastic marker osteopontin was absent in aortas from healthy WT mice and low in Gas6^-/-^ mice (Fig. 5).

#### Older mice

**Fig. 6:**
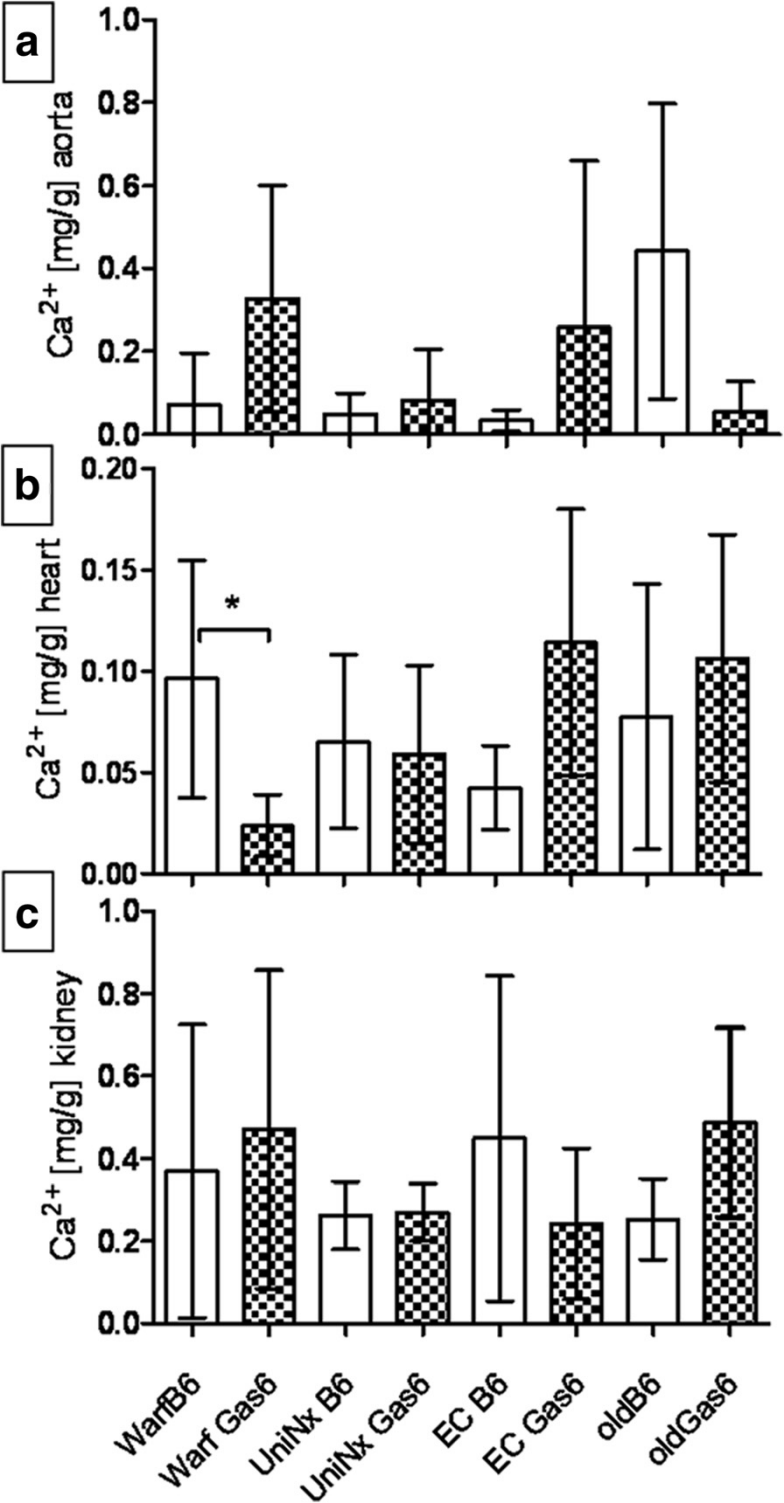
Calcium in tissues; Ca^2+^ content in aortas after warfarin diet, UniNx, EC or in healthy aging WT (C57BL/6) and Gas6^-/-^ mice. **a** aorta, **b** heart, **c** kidney. UniNx (Uninephrectomy), EC (Electrocautery), mean ± SD. –: significant in between groups (*p* < 0.05)

Second, 34–36 week old mice were also assessed. Whereas total serum protein was lower in Gas6^-/-^ mice, all other serum parameters were comparable (Table 1). Calcium content of the aortas, hearts and kidneys was not significantly different between older Gas^-/-^ and WT mice (Fig. 6a-c). Von Kossa staining revealed only minor calcifications in both groups without significant differences. No differences in apoptosis rates in aortas were observed (data not shown).

**Fig. 7:**
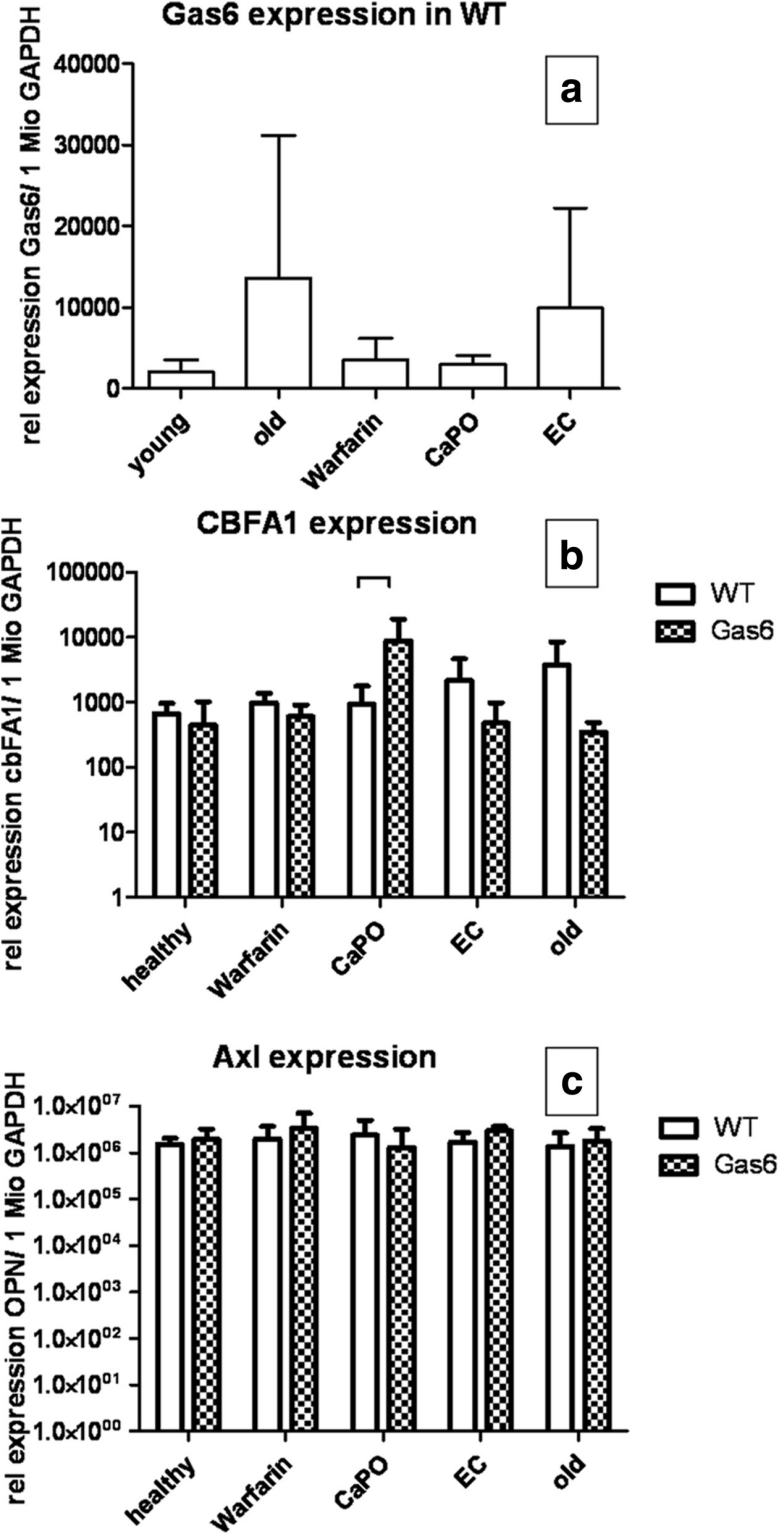
Gene expression; Relative expression of Gas6 in WT (wildtype) (**a**); cbfa1 (**b**) and Axl (**c**) expression in WT and Gas6^-/-^ mice per 1 million copies of GAPDH measured by RT-PCR; mean ± SD

In older Gas6^-/-^ mice MGP gene expression and serum ucMGP level were equal to older WT (Table 1). No differences were found in protein S levels as well (Table 1). There was no difference in Gas6 expression during aging. Cbfa1, Axl and OPN (Fig. 5) expression was similar between Gas6^-/-^ and WT mice (Fig. 7).

### Warfarin treatment

Third, warfarin administration was used to induce calcification by blocking the posttranslational activation of MGP and potentially Gas6. All mice survived warfarin treatment. Serum total protein levels increased significantly in both mouse strains. No significant differences in soft tissue calcification were detected between WT and Gas6^-/-^ mice (Fig. 6a), except for a lower cardiac calcium content in Gas^-/-^ mice after 8 weeks of warfarin (Fig. 6b).

As calcium content in WT mice after warfarin diet was higher only in cardiac tissue compared to Gas6^-/-^ mice, and data from the literature reported that calcification appears in the vicinity of collagen fibres [27] and Gas6 deficiency prevents fibrosis [28] we tested for differences in the cardiac collagen content by Sirius red staining. However, collagen-positive areas in cardiac tissues were equal in warfarin-treated mice in Gas6^-/-^ and WT mice. Serum levels of ucMGP after warfarin diet did not show a significant difference between WT and Gas6^-/-^ mice (Table 2). No differences were found in protein S (Table 2) in serum. No aortic apoptosis could be detected in either of the groups. Cbfa1, Axl and OPN (Fig. 5) expression were similar in Gas6^-/-^ and WT mice (Fig. 7).

**Table 2:** Final biochemistry and echocardiography

Parameter	C57BL\6			Gas6^-/-^		
Model	Warfarin	UniNx	Electrocautery	Warfarin	UniNx	Electrocautery
Body weight [g]	22.9 ± 1.6	24.4 ± 2.2***	22.3 ± 1.6	19.1 ± 1.7	19.1 ± 3.2*	18.5 ± 3.0
*Serum parameter*						
Urea [mmol/l]	6.27 ± 0.63***	10.26 ± 3.58	10.58 ± 1.62	6.43 ± 1.29	10.25 ± 2.56	17.1 ± 11.58
Creatinine [μmol/l]	22.0 ± 1.7	25.1 ± 2.4****	30.3 ± 3.7****	23.6 ± 6.32	29.9 ± 14	30.3 ± 3.8
Calcium [mmol/l]	2.58 ± 0.09	2.50 ± 0.12	2.73 ± 0.14***	2.67 ± 0.17	2.50 ± 0.12	2.84 ± 0.27***
Phosphate [mmol/l]	2.84 ± 0.22	2.78 ± 0.29	2.90 ± 0.30	3.68 ± 0.60	3.47 ± 0.89	3.91 ± 0.68
Protein [g/dL]	6.58 ± 0.38***	5.74 ± 0.74****	5.47 ± 0.49***	6.03 ± 0.32***	5.70 ± 0.31***	5.41 ± 0.55
Alkaline phosphatase [U/l]	205 ± 27	173 ± 65	166 ± 36	228 ± 46	201 ± 64	187 ± 74
ucMGP [nM]	5374 ± 1368	4369 ± 855	3810 ± 1283	5963 ± 2612	4903 ± 1688	4970 ± 489
Protein S [ng/ml]	1.05 ± 0.19	0.80 ± 0.12	0.72 ± 0.03	0.83 ± 0.24	0.83 ± 0.23	0.83 ± 0.27
*Echocardiography*						
Left ventricular mass [mg]	79.0 ± 22	60.7 ± 15	59.1 ± 12	69.2 ± 14.7	74.1 ± 28.5	61.4 ± 16.6
Stroke volume [μl]	22.0 ± 6.0	13.4 ± 2.7	10.4 ± 2.8	20.6 ± 4.3	24.4 ± 8.9	10.2 ± 6.2
Ejection Fraction [%]	46.6 ± 11.6	33.3 ± 13.8	33.7 ± 7.4	44.8 ± 2.6	58.4 ± 21*	38.4 ± 18
VACC [mm/ms]	1.97 ± 0.71	2.69 ± 1.01	1.78 ± 0.46	2.26 ± 2.14	3.03 ± 1.38	1.98 ± 0.37
*24 h urine*						
GFR [μL/min]	76.9 ± 36.9	55.2 ± 32.4	41.7 ± 21.3	56.6 ± 32.8	41.6 ± 28.1	40.4 ± 13.0
Protein [mg/dl]	26. ± 17	13 ± 4.6***	6.7 ± 1.9***	14.0 ± 4.9	9.5 ± 6***	17.7 ± 4.2
Creatinine [μmol/l]	2071 ± 2005	3853 ± 2520	3062 ± 1133	4283 ± 3853	2175 ± 667	914 ± 179

*p < 0.05 to corresponding wildtype group *** *p* < 0.05 to healthy control group ****p < 0.001 to healthy control group

Biochemical and functional characteristics of wildtype and Gas6^-/-^ after 8 weeks of treatment (Mean ± SD), *GFR* glomerular filtration rate, *ucMGP* uncarboxylated matrix Gla protein, *UniNx* uninephrectomy, *VACC* pulse-wave velocity over the arteria carotis communis)

### Uninephrectomy

**Fig. 8:**
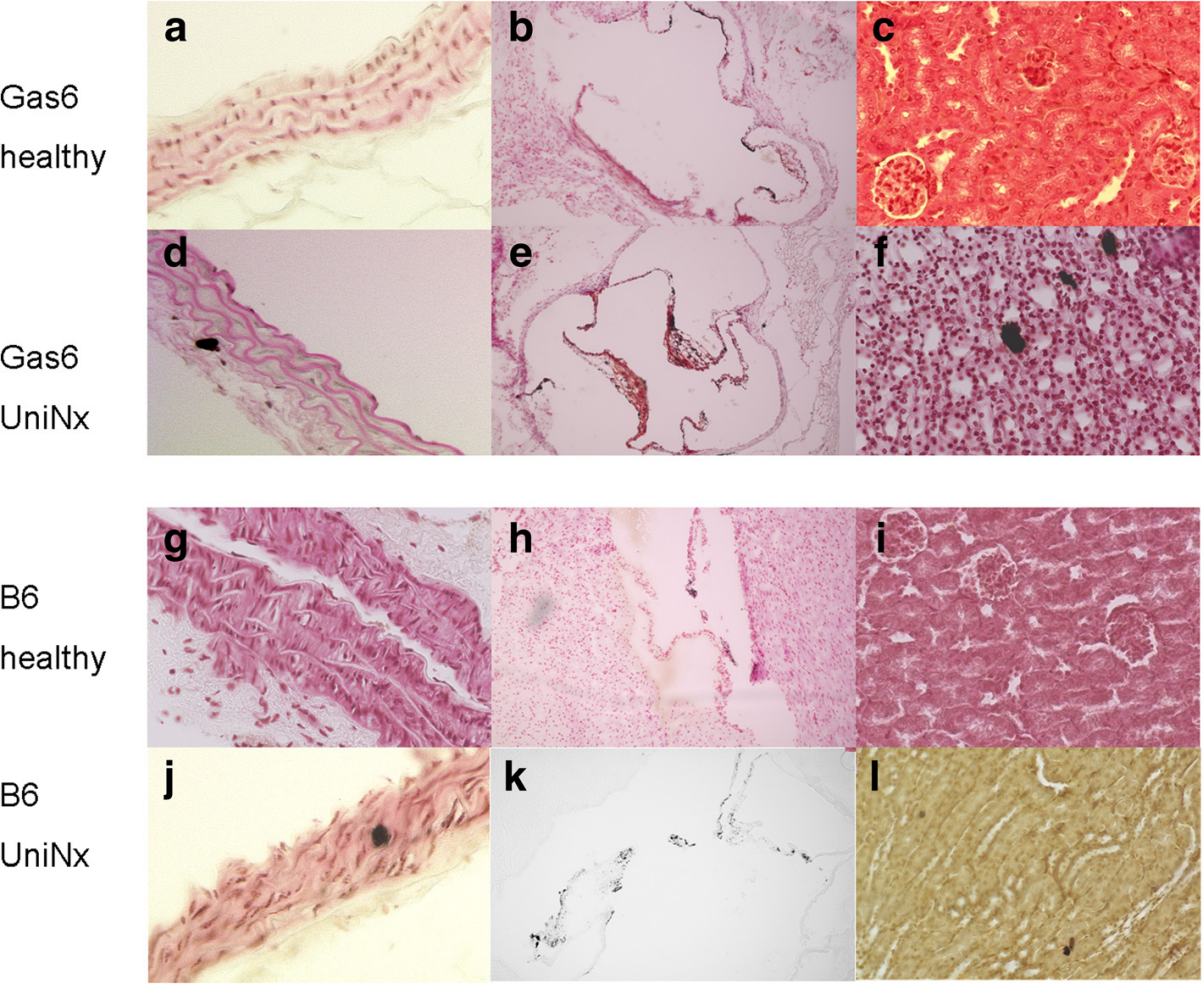
Localisation of calcium deposits; *Von Kossa* staining of the aorta (**a**, **d**, **g**, **j**), heart (**b**, **e**, **h**, **k**) and kidney (**c**, **f**, **i**, **l**) after uninephrectomy in WT (Wildtype) compared to Gas6^-/-^ mice. WT; Magnification 400x

Fourth, UniNx (plus high phosphate diet) was used to initiate kidney damage. In both UniNx groups, 15–18 % of the mice died. Early death occurred in 2 of 13 in the Gas6^-/-^ mice during anaesthesia and in 2 of 11 WT mice. In WT mice one animal died during surgery and the other one 3 weeks after surgery. Serum creatinine increased after surgery, compared to healthy animals, in Gas6^-/-^ and WT mice (+46 % in WT and +24 % in Gas6^-/-^, respectively) (Table 2). Spotty calcification was present in the aorta (Fig. 8a) and in cardiac tissue, however, without obvious differences between Gas6^-/-^ and WT mice (Fig. 6b). Within the heart, calcifications mainly located to the valves (Fig. 8b). The most prominent calcification was found in kidney tissues in both strains, spreading throughout all structures with accumulation in the renal cortex (Fig. 8c). Again, there was no significant difference between the two mouse strains with respect to tissue calcium content (Fig. 6a-c).

Echocardiography after UniNx revealed decreased ejection fractions in WT compared to Gas6^-/-^ (Table 2). Ejection fraction and stroke volume were lower in both strains than in healthy controls. No aortic apoptosis could be detected in both groups. In Gas6^-/-^ mice MGP gene expression, serum ucMGP and protein S levels were equal to WT (Table 2). Cbfa1 expression significantly increased in Gas6^-/-^ compared to WT mice, however this could not be observed in the electrocautery group. Axl and OPN (Fig. 5) expression were similar between Gas6^-/-^ and WT mice (Fig. 7).

### Electrocautery

**Fig. 9:**
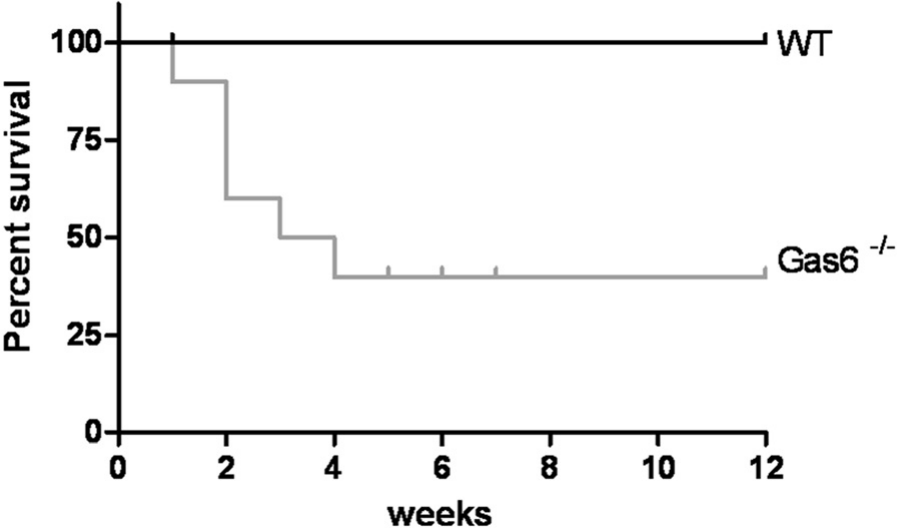
Survival; Kaplan-Meier curve after electrocautery surgery in WT (Wildtype) and Gas6^-/-^mice

Finally, electrocautery of the contralateral kidney was used in addition to UniNx (plus high phosphate diet) to further increase the extent of kidney damage. In the electrocautery group, all WT mice survived the surgical protocol, however, the Gas6^-/-^ mice, showed decreased survival without detectable change in body weight relatively early after surgery, and only 40 % survived to the end of the experiment (Fig. 9), thus all data below have to be interpreted with this caveat in mind. No obvious cause for the high mortality could be identified.

Serum calcium, phosphate and creatinine were significantly increased in WT and surviving Gas6^-/-^ mice after electrocautery compared to the corresponding healthy controls (Table 2). Calcium content in the aortas and hearts of Gas6^-/-^ mice was slightly higher than in WT, however, without a significant difference (Fig. 6). Calcification spots were found in a similar pattern as after UniNx in all kidney structures in both WT and Gas6^-/-^ mice.

Electrocautery decreased ejection fraction and stroke volume only significantly in WT mice (Table 2). No relevant aortic apoptosis could be detected in either of the groups. In Gas6^-/-^ mice aortic MGP gene expression (Table 2), serum ucMGP and protein S level were equal to WT (Table 2). Cbfa1, Axl and OPN (Fig. 5) expression were similar in Gas6^-/-^ and WT mice (Fig. 7).

## Discussion

Gas6 has repeatedly been suggested to mediate vitamin K effects in vascular calcification in addition to MGP. To evaluate the role of Gas6 protein in vascular calcification, we compared mice deficient in Gas6 protein with WT mice. VSMC and mice were exposed to different calcification and kidney disease models.

The major finding of this study is that both in vitro and in vivo vascular as well as organ calcification of WT and Gas6^-/-^ mice were not different. Previously, Gas6 mRNA stabilisation was found to be a protective mechanism of statins in cell culture experiments [16]. In these in vitro experiments, Gas6 mediated protection against calcification. However, these findings were obtained only in vitro and in contrast to the present study, no in vivo verification was attempted.

Transformation of VSMC into an osteoblastic phenotype is a highly regulated process. Among others, the vitamin K-dependent protein MGP [29] and apoptosis, in particular apoptotic bodies [30, 31], have been shown to play important roles in the calcification process. The vitamin K-dependent protein Gas6 reportedly mediates anti-apoptotic effects [15] and could thereby conceivably prevent calcification like MGP. However, here we failed to observe increased apoptosis in Gas6^-/-^ mice in vivo and in vitro when compared to WT mice and we thus cannot confirm a role of Gas6 in an anti-apoptotic pathway in our uremia and calcification models.

To test for other potential counterregulatory mechanisms against calcification in the Gas6^-/-^ mice that might have compensated for the lack of Gas6 functions, we analyzed MGP gene expression, ucMGP staining in the aorta and ucMGP serum levels. MGP is known to be the most important local calcification inhibitor in the vessel wall [7]. MGP, like Gas6, is vitamin K dependent for bioactivity and could act as a compensatory mechanism for the lack of Gas6. However, MGP was neither upregulated at the mRNA level in the aortic wall nor did we find increased differences between Gas6^-/-^ and WT mice with respect to circulating ucMGP serum levels. Axl expression, an inhibitor of apoptosis and thus vascular calcification, was also unaltered in Gas6^-/-^ mice. Its ligand besides Gas6, protein S was equal in all our in vivo groups and therefore seems unlikely to act as a counterregulator in our models.

Interestingly, Gas6^-/-^ mice exhibited a higher left ventricular mass despite a lower body weight. This might also partially explain why the ejection fraction after surgery in WT was significantly lower than in Gas6-/- mice (Tables 1 and 2).

Strikingly, calcium content of the myocardium was significantly lower in Gas6^-/-^ mice compared to WT after warfarin diet. Chemical quantification and *von Kossa* staining revealed only weak total myocardial calcification in Gas6^-/-^ and WT. Cardiac calcification is also associated with fibrosis [32] and Gas6 deficiency has been shown to prevent fibrosis [28]. Therefore, we speculate that protective effects of lack of Gas6 might depend on a different collagen content of the hearts. However, in our study, cardiac collagen staining was equal in both Gas6 and WT mice.

We observed a reduced survival of the knockout mice after electrocauterization plus UniNX compared to WT. As the mice died at different time points close to the operation procedures, calcification processes appear unlikely to be the cause of premature death. One can speculate that inflammatory processes may play a role. For example, Gas6 secretion is stimulated by TGF-ß [33] and it was found to be expressed in atherosclerotic plaques by VSMC and negatively associated with inflammation [33]. In our experiments, however, we observed neither signs of inflammation in the vessel tissue nor accumulation of collagen fibers in Sirius red staining. Gas6 protein was shown to enhance interactions of endothelial cells and leukocytes. Inflammation as a cause seems unlikely, as leukocyte infiltration is reduced in Gas6^-/-^ animals [34]. Other potential reasons could play a role in the decreased survival rate. The initial slightly lower body weight of the knockouts might hamper their survival after electrocautery. Alternatively, Gas6 knockout mice were reported to display platelet dysfunction [35] and Gas6 protein contributes to thrombus formation [23]. However, altered coagulation of the uremic Gas6^-/-^ mice are again unlikely to have contributed to increased mortality, as we could not find evidence for hemorrhage or infarctions. Confirmatory to our results, Axl^-/-^ mice were recently found to have a reduced survival after renal mass reduction and high phosphate diet, but significant vascular calcification was not induced. Axl activation might reduce the progression of tubulo-interstitial apoptosis [24] and activates several cell survival factors [12].

One potential limitation of this study is the C57BL/6 genetic background of the mice. Black six mice calcify to a lesser extent than DBA/2 mice [36, 37]. With additional trigger, i.e. on top of nephrectomy, calcification in B6 mice is possible. Mice lacking fetuin A on a black six background (B6,129-Ahsgtm1Mbl) develop ectopic microcalcifications in soft tissues [25, 26, 38] and only when backcrossed onto a DBA/2 background, does severe calcification develop [25]. Again, warfarin induced remarkable calcification in DBA/2 mice [39]. Nevertheless, in all of our models, minor calcification could be induced and the lack of aggravated calcification by depletion of Gas6 does not support a major role in vascular calcification pathogenesis.

## Conclusion

Taken together, we were not able to prove a prominent role of Gas6 in vascular calcification. Further studies should confirm the role of Gas6 in disease models.

## Abbreviation

BGP, Beta glycerol phosphate; EF, Ejection fraction; KH2, Vitamin K hydroquinone; LV, Left ventricular; MGP, Matrix gla protein; OPN, Osteopontin; PWV, Pulse wave velocity; ucMGP, Uncarboxylated matrix gla protein; VSMC, Vascular smooth muscle cells; WT, Wildtype.
